# Impact of external pneumatic compression target inflation pressure on transcriptome‐wide RNA expression in skeletal muscle

**DOI:** 10.14814/phy2.13029

**Published:** 2016-11-24

**Authors:** Jeffrey S. Martin, Wesley C. Kephart, Cody T. Haun, Anna E. McCloskey, Joshua J. Shake, Christopher B. Mobley, Michael D. Goodlett, Andreas Kavazis, David D. Pascoe, Lee Zhang, Michael D. Roberts

**Affiliations:** ^1^ Department of Cell Biology and PhysiologyEdward Via College of Osteopathic Medicine – Auburn CampusAuburnAlabama; ^2^ School of KinesiologyAuburn UniversityAuburnAlabama; ^3^ Athletics DepartmentAuburn UniversityAuburnAlabama; ^4^ Department of Entomology and Plant PathologyAuburn UniversityAuburnAlabama

**Keywords:** External pneumatic compression, PGC‐1*α*, redox balance, RNA sequencing, skeletal muscle

## Abstract

Next‐generation RNA sequencing was employed to determine the acute and subchronic impact of peristaltic pulse external pneumatic compression (PEPC) of different target inflation pressures on global gene expression in human *vastus lateralis* skeletal muscle biopsy samples. Eighteen (*N* = 18) male participants were randomly assigned to one of the three groups: (1) sham (*n* = 6), 2) EPC at 30–40 mmHg (LP‐EPC;* n* = 6), and 3) EPC at 70–80 mmHg (MP‐EPC;* n* = 6). One hour treatment with sham/EPC occurred for seven consecutive days. *Vastus lateralis* skeletal muscle biopsies were performed at baseline (before first treatment; PRE), 1 h following the first treatment (POST1), and 24 h following the last (7th) treatment (POST2). Changes from PRE in gene expression were analyzed via paired comparisons within each group. Genes were filtered to include only those that had an RPKM ≥ 1.0, a fold‐change of ≥1.5 and a paired *t*‐test value of <0.01. For the sham condition, two genes at POST1 and one gene at POST2 were significantly altered. For the LP‐EPC condition, nine genes were up‐regulated and 0 genes were down‐regulated at POST1 while 39 genes were up‐regulated and one gene down‐regulated at POST2. For the MP‐EPC condition, two genes were significantly up‐regulated and 21 genes were down‐regulated at POST1 and 0 genes were altered at POST2. Both LP‐EPC and MP‐EPC acutely alter skeletal muscle gene expression, though only LP‐EPC appeared to affect gene expression with subchronic application. Moreover, the transcriptome response to EPC demonstrated marked heterogeneity (i.e., genes and directionality) with different target inflation pressures.

## Introduction

External pneumatic compression (EPC) therapies vary widely in their target inflation pressures (e.g., 10–300 mmHg) due, in part, to the numerous maladies or functions they are specifically designed to target. For example, enhanced external counterpulsation (EECP), a therapy traditionally used for treatment of coronary artery disease patients, utilizes target inflation pressures of up to 300 mmHg on the lower limbs, whereas intermittent pneumatic compression (IPC), used for the treatment of venous insufficiency, lymphedema, etc., utilizes inflation pressures of 20–120 mmHg on the lower limbs.

Peristaltic pulse EPC (PEPC) is similar to IPC in that compression is applied to the lower limbs with lower target inflation pressures (20–100 mmHg), though it differs in several ways including, but not limited to, the duty cycle and peristaltic nature of the compressive stimulus. We have previously reported that 35 one hour sessions of EECP evoked significant changes in skeletal muscle capillarity and protein expression (i.e., GLUT‐4) (Martin et al. 2012). Moreover, we recently demonstrated that a single 1 h bout of PEPC with target inflation pressures of 70 mmHg significantly alters gene expression of inflammation‐, redox‐, hypoxia‐, and metabolism‐associated genes in skeletal muscle (Kephart et al. 2015). These findings were in accordance with others who have demonstrated acute alteration of skeletal muscle gene expression in both human (Sheldon et al. 2012; Roseguini et al. 2010) and animal models (Tan et al. 2006) with lower pressure (pressures ranged from 55 to 120 mmHg) EPC therapy. Thus, the evidence supports EPC‐mediated alterations in skeletal muscle gene expression though, to the best of our knowledge, only acute investigations with lower pressure (≤120 mmHg) EPC interventions have been performed to date.

Of note, our previous investigations exploring the molecular effects of PEPC utilized target inflation pressures of 70 mmHg given the beneficial effects seen with this pressure in vascular and sports performance studies (Martin et al. 2015a,b). However, the effect of lower target inflation pressures with this PEPC device is unknown. Others have suggested that the frequency of compression (i.e., duty cycle), and not magnitude, was a primary factor mediating the significant alteration in gene expression (Sheldon et al. 2012; Roseguini et al. 2010). However, similar to our previous investigations, a targeted approach toward an organ/system of interest (e.g., vascular function, inflammation) was used to determine the change in selected targeted genes with EPC, and these responses were only assessed following a single EPC bout. In this regard, it is plausible that significant alteration of nontargeted genes was not “captured” by this targeted, acute approach and that EPC‐mediated alteration of gene expression is indeed pressure‐sensitive.

Thus, the purpose of the present investigation was to characterize the acute and subchronic effects of PEPC on skeletal muscle gene expression across the entire transcriptome at different target inflation pressures in humans using RNA sequencing and western blotting confirmation of the sequencing results. Additionally, we sought to localize (e.g., vascular vs. muscular) select PEPC‐mediated alterations in molecular markers based on previous findings (e.g., nuclear localization of PGC‐1*α*, phosphorylation of eNOS) (Kephart et al. 2015). Based upon the marked heterogeneity of gene transcription responses previously observed with different stimuli (exercise type, obesity, pharmacological intervention, etc.) in a single tissue type (vasculature) (Jenkins et al. 1985; Laughlin et al. 2015a,b; Padilla et al. 2014a, 2013,b), we hypothesized that there would be differential gene transcription responses in skeletal muscle biopsy samples with distinct PEPC target inflation pressures. Specifically, and based upon the previous literature, we hypothesized that gene expression related to inflammation, redox handling, and/or hypoxia would be altered in a dose‐dependent manner. Finally, we hypothesized that the subchronic PEPC‐mediated changes in gene expression would demonstrate heterogeneity with that observed acutely.

## Methods

### Ethical approval

All procedures described herein were approved by Auburn University's Institutional Review Board and conformed to the standards set by the latest revision of the Declaration of Helsinki. In addition, written informed consent was obtained from all participants prior to their voluntary participation in the study.

### Participants

Eighteen (*N* = 18) college‐aged, male volunteers were recruited for participation in this study. Eligibility criteria included the following: (1) participants were apparently healthy, medication‐free, recreationally active, and had no history of blood clotting issues; and (2) participants had not consumed ergogenic nutritional supplements for at least 1 month prior to enrolment. Participants also filled out a short questionnaire indicating their self‐reported amounts of weekly physical activity (lower body resistance training and endurance exercise training). After enrolment, subjects were randomly assigned to one of the three groups: sham (*n* = 6), low pressure PEPC (LP‐PEPC; 30–40 mmHg; *n* = 6), and moderate pressure PEPC (MP‐PEPC; 70–80 mmHg; *n* = 6).

### Compression protocol and skeletal muscle biopsies

All participants enrolled in the study reported to the laboratory for eight consecutive days. On the date of the first visit, participants were instructed to report to the laboratory (day 1) following 4 h devoid of food and/or caffeine‐containing beverages and at least 24 h being refrained from rigorous physical activity and alcohol and tobacco use. Participants were then instructed to lay in a supine position on a treatment table, whereby a pretreatment percutaneous skeletal muscle biopsy was obtained from the left *vastus lateralis* midway between the patella and iliac crest using a 5 gauge needle with suction and sterile laboratory procedures (termed PRE biopsy). Briefly, 1.5 mL of 1% lidocaine was injected subcutaneously above the skeletal muscle fascia prior to making a small pilot incision for needle intrusion. The biopsy needle was then inserted at a depth just beyond the fascia, and approximately, 100–150 mg of skeletal muscle was removed using a double‐chop method and applied suction. Extracted tissue was immediately blotted of visible blood using sterile gauze pads and had all visible fat and connective tissue removed. Thereafter, tissue processing occurred as follows: (1) approximately, 50 mg tissue was immediately placed in a 1.7 mL polypropylene tube containing 500 μL of cell lysis buffer (Cell Signaling, Danvers, MA) spiked with 1x phosphatase II and III inhibitors (G Biosciences, Saint Louis, MO) and processed for protein analyses as described below, (2) 10–20 mg of muscle was placed in a 1.7 mL polypropylene tube containing 500 μL of Ribozol (Amresco, Solon, OH) for mRNA analyses as described below, (3) ~100 mg was placed in a small plastic tissue molder, slow‐frozen in liquid nitrogen‐cooled isopentane in optimal cutting temperature (OCT) media, and subsequently stored at −80°C for immunohistochemistry analyses described below, and (4) remaining tissue was snap‐frozen in liquid nitrogen and subsequently stored at −80°C.

After the initial skeletal muscle biopsy, sham, LP‐PEPC, or MP‐PEPC was applied to the lower limbs for 1 h according to group assignments. The EPC device utilized herein (NormaTec, Newton, MA) consists of two separate “leg sleeves” which contain five circumferential inflatable chambers (arranged linearly along the limb) encompassing the leg from the feet to the hip/groin. The “leg sleeves” are connected to an automated pneumatic pump at which target inflation pressures for each zone and the duty cycle can be controlled. In this study, we chose to use inflation protocols consisting of low (30–40 mmHg) and moderate (70–80 mmHg) target inflation pressures for each chamber. We have previously investigated and described MP‐PEPC (Kephart et al. 2015; Martin et al. 2015a,b). LP‐PEPC is essentially the same, only with different target inflation pressures. In brief, beginning with the most distal chamber, inflation occurs and the chamber “pulses” (slight variations in pressure) for 30 sec after which pressure is held constant to prevent backflow. The same process then occurs in each of the next highest zones individually. Notably, a maximum of only two distal chambers is held at constant pressure to facilitate greater rest time (no compression) in each chamber. After the most proximal zone completes its cycle, all zones are completely deflated for 30 sec. This entire cycle of compression is repeated continuously over the course of a single 1‐h treatment session (20 complete cycles). The sham treatment condition consisted of 1‐h application of the EPC “leg sleeves” and connection to the pneumatic pump, without actual compression.

Following the initial 1‐h sham or PEPC protocol, the leggings were removed and subjects passively rested on a treatment table. At 1 h following the completion of the 1‐h sham or PEPC treatment, a skeletal muscle biopsy was obtained from the right *vastus lateralis* in order to reduce the potential inflammatory effects that could have carried over from the PRE‐EPC biopsy (termed POST1 biopsy).

Participants were dismissed following the 1‐h posttreatment biopsy and instructed to refrain from rigorous physical activity and to not deviate from normal dietary habits during the duration of the study. Participants reported to the laboratory for six additional, consecutive days (days 2–7) for treatment with sham or PEPC according to their group assignment. Twenty‐four hours following the last sham or PEPC session, participants again reported to the laboratory (day 8) following 4 h devoid of food and/or caffeine‐containing beverages and at least 24 h being refrained from rigorous physical activity and alcohol and tobacco use. At this time, a final skeletal muscle biopsy was harvested from the right *vastus lateralis* (termed POST2 biopsy). This biopsy was obtained more proximal to the 1‐h post‐PEPC biopsy sample due to prior literature showing this sampling sequence prevents inflammatory signaling events that may occur with a two‐site biopsy approach (Van Thienen et al. 2014).

### RNA isolation and preparation for sequencing

About 10–20 mg portions of muscle biopsy samples were homogenized in 500 μL of Trizol (Amresco) and stored at −80°C for batch processing. During batch processing, total RNA was isolated according to manufacturer's instructions. RNA was then further purified using RNA isolation columns (RNA easy Micro Kit; Qiagen Inc, Valencia, CA) according to manufacturer's instructions. Thereafter, RNA concentrations were assessed using a NanoDrop Lite (Thermo Scientific, Waltham, MA) prior to cDNA synthesis for mRNA analyses. RNA concentrations of 500 ng were subsequently shipped to the Auburn University Genomics and Sequencing Laboratory for RNA deep sequencing. First, high RNA integrity of each sample was confirmed (RIN > 7.0) using a bioanalyzer 2100 automated electrophoresis system (Agilent Technologies, Santa Clara, CA) prior to cDNA library constructions. Thereafter, cDNA libraries were constructed using Illumina's TruSeq Stranded mRNA Sample Preparation Kit (Illumina, San Diego, CA) and library quantification was performed using the Kapa quantification kit for next‐generation sequencing with the Illumina platform (Kapa Biosystems, Wilmington, MA). Each sample of 12 pmol/L concentrations was loaded into flowcells, where clusters of each oligo was replicated. Eight samples were loaded per lane and randomly distributed within and across lanes. The flowcells were placed in the HiSeq 1500 sequencer (Illumina Inc) and fluorescently labeled bases were attached to the complementary bases of each sequence. Next‐generation sequencing was performed with 50 bp single read single indexing sequencing protocol in Rapid Mode. Sequencing data were demultiplexed with CASAVA software 1.8.2 (Illumina).

Following sequencing, reads were trimmed to ensure adaptor sequence removal and tiled to a human transcriptome database using NextGENe v2.2 software (SoftGenetics, State College, PA). Reads per kilobase of exon per million reads mapped (RPKM) values were assigned to each transcript. RPKM is a value that quantifies gene expression from DNA sequencing data while normalizing to the number of sequence reads and total read length (Mortazavi et al. 2008) and is the value that is used to compare transcript expression within‐groups.

### Transcript filtering and bioinformatics

At present, there is no universally accepted standard for RNA‐seq bioinformatics. Thus, the RNA‐seq literature was examined and we devised an approach for a modified filtering process based on other current filtering procedures (Song et al. 2012; Roberts et al. 2013; Toedebusch et al. 2014; Ruegsegger et al. 2015). All bioinformatics procedures were performed with Microsoft Excel v2013 (Microsoft Corporation, Redmond, WA). Since we were interested in within‐subject changes, and cannot rule out any effect of the sham condition, our analyses were focused on within‐treatment changes in gene transcription relative to baseline. Thus, PRE versus POST1 and PRE versus POST2 comparisons were made within each treatment group. Briefly, our bioinformatics procedure included the following: (1) omitting transcripts with unknown functions and those with an average RPKM value across all samples of <1.0, (2) omitting transcripts where average fold‐change from PRE was <1.5 for all within‐group comparisons, and (3) removing transcripts when within‐subject Student's paired *t*‐tests (i.e., PRE vs. POST1, PRE vs. POST2) had a *P*‐value of ≥0.01 for all comparisons.

Of the 25 579 annotated genes identified in our data set, 3 610 had an average RPKM value across all samples of ≥1.0. Filtering for transcripts where a fold‐change from PRE of greater than 1.5 for any comparison resulted in 2566 remaining transcripts. Finally, of the remaining transcripts, 71 were uniquely identified as significant within‐subject changes from PRE via a Student's paired *t*‐tests (*P*‐value of ≤0.01).

### Ingenuity pathway analysis

The generated lists of significantly altered genes (i.e., 71 transcripts) and the respective conditions were further investigated using Ingenuity Pathway Analysis (IPA; Ingenuity Systems, http://www.ingenutiy.com). First, the IPA software was used for functional analyses to identify significantly altered (based on Fisher's exact test) biological processes. Second, IPA was used to generate gene networks that were scored based on the number of Network Eligible Molecules (NEMs) they contained, so the higher the score, the lower the probability of finding the observed number of NEMs in a given network by random chance. The score itself is calculated as the negative log10 of the *P*‐value from Fisher's exact test applied to a given network. Finally, we employed upstream regulator analysis in IPA in order to determine upstream molecules in the causal network (Ingenuity Knowledge Base) that are connected to the significantly affected genes herein. Fisher's exact test is used to identify significant upstream regulators and an activation score is calculated based on the concordance of the predicted direction of the causal relationships, with each relationship weighted according to the degree of consensus in the literature and the number of reported findings they are based upon. The final score is reported in terms of z‐scores.

### Protein isolation and western blotting

Immediately following muscle extraction, samples were homogenized using a tight‐fitting micropestle, insoluble proteins were removed with centrifugation at 500 × *g* for 5 min at 4°C, and supernatants containing muscle tissue homogenate were collected and stored at −80°C. After all participants finished the study, the muscle tissue homogenates were batch‐assayed for total protein content using a BCA Protein Assay Kit (Thermo Scientific, Waltham, MA).

Cell lysis homogenates obtained from above were prepared for western blotting using 4x Laemmli buffer at 1 μg/μL. Thereafter, 20 μL of prepped samples were loaded onto 12% SDS‐polyacrylamide gels (BioRad, Hercules, CA) and subjected to electrophoresis (180 V at 60 min) using premade 1x SDS‐PAGE running buffer (Amresco). Proteins were then transferred to polyvinylidene difluoride membranes (BioRad), Ponceau stained, and imaged to ensure equal protein loading between lanes. Thereafter, membranes were blocked for 1 h at room temperature with 5% nonfat milk powder. Rabbit anti‐4HNE IgG (1:1,000; Abcam, ab46545, Cambridge, MA), anti‐PGC‐1*α* (1:1,000; Santa Cruz, sc‐13067, Dallas, TX), anti‐VEGF (1:1,000; Abcam, ab115961), anti‐phospho‐AMPK (Thr172) (1:1,000; Cell Signaling, #2535), anti‐catalase (1:1,000; GeneTex, GTX110704, Irvine, CA), and anti‐GAPDH (1:1,000; GeneTex, GTX100118) were incubated with membranes overnight at 4°C in 5% bovine serum albumin (BSA), and the following day, membranes were incubated with horseradish peroxidase‐conjugated anti‐rabbit IgG (1:2,000, Cell Signaling, #7074) at room temperature for 1 h prior to membrane development. Membrane development was performed using an enhanced chemiluminescent reagent (Luminata Forte HRP substrate; Millipore, Billerica, MA), and band densitometry was performed through the use of a gel documentation system and associated densitometry software (UVP, Upland, CA). Of note, the densitometry values for protein targets were normalized to GAPDH densities.

### Immunohistochemistry

Sections from OCT‐preserved samples were cut at a thickness of 10 μm using a cryotome (HM 525 Cryostat; Thermo Fisher Scientific, Waltham, MA) and were adhered to positively charged histology slides. Once all samples were sectioned, batch processing occurred for immunohistochemistry. Briefly, sections were dried at room temperature for 30 min and incubated in a phosphate‐buffered saline (PBS) solution containing 0.5% Triton X‐100. Sections were rinsed in PBS and were immunostained as follows: (1) for phosphorylated eNOS detection in capillaries, a cocktail of mouse anti‐dystrophin IgG (1:100; Abcam, ab14451) and rabbit anti‐phospho‐eNOS (Ser1177) IgG (1:100; Abcam, ab184154) in PBS containing 5% blocking solution (Super Blocker; Thermo Fisher Scientific) was used; (2) for PGC‐1*α* detection in myofibers and nuclei, a cocktail of rabbit anti‐PGC‐1*α* IgG (1:100; Abcam, ab54481) and mouse anti‐MAB IgG (1:100; Abcam, ab24609) in PBS containing 5% blocking solution (Super Blocker; Thermo Fisher Scientific, #37535) was used. Immunostaining with respective primary antibodies occurred over 1 h. Thereafter, slides were rinsed in PBS and incubated for 1 h with a cocktail containing goat anti‐rabbit IgG (Texas Red‐conjugated) (1:100; Vector Laboratories, T1‐1000, Burlingame, CA) and anti‐mouse IgG (FITC‐conjugated; Santa Cruz, sc‐2010). Thereafter, slides were rinsed in PBS, mounted with glass coverslips and DAPI media (Vector Laboratories, H‐1200), and stored in the dark until imaging.

Imaging occurred whereby the tester was blinded to which treatment group the subject was assigned. For each subject, and at each time point, three 40x images of each respective fluorescent filter [capillaries: Texas Red (phospho‐eNOS) and FITC (dystrophin); PGC‐1*α* localization: DAPI (nuclei), MAB (nuclei), and PGC‐1*α* (Texas Red)] were obtained using a fluorescent microscope (Nikon Eclipse Ti‐U; Nikon Instruments, Melville, NY). Images were merged using the NIS Elements software (Nikon), the number of capillaries that contained phospho‐eNOS within a field of image were quantified, and percent of myonuclei that contained PGC‐1*α* were quantified.

### Citrate synthase activity

Citrate synthase activity in the skeletal muscle biopsy samples was performed according to the methods of Trounce et al. 1996. The citrate synthase activity assay utilized is based on the reduction of 5,5′‐dithiobis(2‐nitrobenzoic acid) (DTNB) at 412 nm (extinction coefficient 13.6 mmol/L^−1^ cm^−1^) coupled to the reduction of acetyl‐CoA by the citrate synthase reaction in the presence of oxaloacetate. Briefly, 50 μg of skeletal muscle protein were added to a mixture composed of 0.125 mol/L Tris‐HCl (pH 8.0), 0.03 mmol/L acetyl‐CoA, and 0.1 mmol/L DTNB. The reaction was initiated by the addition of 5 μL of 50 mmol/L oxaloacetate and the absorbance change was recorded for 1 min.

### Statistics

The reader is referred above for statistical analyses methods pertaining to filtering of RNA sequencing data and identification of significant within‐treatment group changes in gene transcription. Between‐group subject characteristics were compared using a one‐way analysis of variance (ANOVA) with group assignment as the between‐group factor. Changes in protein expression values derived from western blotting and immunohistochemistry were evaluated using repeated measures ANOVA. When a significant group × time interaction was observed (*P ≤ *0.05), post hoc analysis was performed using paired *t*‐tests to determine significant within‐group changes from PRE. In these instances, Bonferroni correction for multiple comparisons was applied and an alpha level ≤0.025 was required for statistical significance. In addition, given the relatively small sample sizes, for western blotting and IHC investigations, effect sizes (ES) were also calculated and reported in instances at which a large ES was observed according to Cohen's guidelines (ES > 0.80) (Cohen 1992). Effect sizes were calculated as the ratio of the mean difference between respective post‐PEPC time points and PRE to the standard deviation of difference between the respective time points. All statistical analyses were performed using IBM SPSS Statistics 24 for Windows (Chicago, IL) and Microsoft Excel 2013 (Redmond, WA).

## Results

Participant characteristics are presented in Table 1. No significant differences between groups were found for age, height, weight, BMI, resistance training, endurance training, and total training (*P *>* *0.05 for all).

**Table 1:** Subject characteristics

Group	Age (years)	Height (m)	Weight (kg)	BMI (kg/m^2^)	RT (h/week)	ET (h/week)
Overall (*N* = 18)	23.6 ± 0.7	1.80 ± 0.02	91.5 ± 3.03	28.1 ± 0.9	1.3 ± 0.2	2.4 ± 0.2
Sham (*n* = 6)	22.9 ± 0.7	1.85 ± 0.02	98.7 ± 4.6	28.9 ± 1.5	1.2 ± 0.4	2.4 ± 0.3
LP‐PEPC (*n* = 6)	22.4 ± 0.5	1.77 ± 0.03	86.2 ± 5.8	27.6 ± 1.8	1.2 ± 0.5	2.5 ± 0.2
MP‐PEPC (*n* = 6)	25.5 ± 1.6	1.80 ± 0.02	89.9 ± 4.8	27.8 ± 1.6	1.5 ± 0.4	2.4 ± 0.3

BMI, body mass index; RT, lower body resistance training; ET, endurance training; LP‐PEPC, low‐pressure peristaltic pulse external pneumatic compression with target inflation pressures of 30–40 mmHg; MP‐PEPC, moderate pressure PEPC with target inflation pressures of 70–80 mmHg.

### Transcriptome‐wide changes in gene expression with LP‐PEPC and MP‐PEPC

A full list of significantly altered genes relative to PRE across all conditions at the POST1 time point is presented in Table 2. There were two genes identified that changed significantly from PRE to POST1 in the sham treatment group (up‐regulation of *FIS1* and *SF3B5*). There were nine and 23 genes that changed significantly from PRE to POST1 in the LP‐PEPC and MP‐PEPC treatment groups, respectively. For the LP‐PEPC condition, all eight genes were up‐regulated, whereas 21 of the 23 significantly affected genes were down‐regulated in the MP‐PEPC group.

**Table 2:** Relative changes in gene expression from PRE 1 h following a single 1 h bout of sham, low pressure (LP‐PEPC; 30–40 mmHg), and moderate pressure (MP‐PEPC; 70–80 mmHg) peristaltic pulse external pneumatic compression (POST1 time point)

Gene	Description	Fold‐change from PRE		
		Sham	LP‐PEPC	MP‐PEPC
Genes up‐regulated relative to PRE				
FIS1	Fission, mitochondrial 1	**1.559** *	1.043	1.016
SF3B5	Splicing factor 3b subunit 5	**1.509** *	1.161	1.223
HIST1H2AC	Histone cluster 1, H2ac	2.271	**3.889** *	1.897
ARRDC2	Arrestin domain containing 2	1.786	**3.207** *	−1.042
DYNLL2	Dynein, light chain, LC8‐type 2	1.912	**2.010** *	−1.079
SRXN1	Sulfiredoxin 1	1.743	**1.845** *	−1.145
HNRNPA0	Heterogeneous nuclear ribonucleoprotein A0	1.333	**1.676** *	1.015
RSL24D1	Ribosomal L24 domain containing 1	1.419	**1.646** *	−1.355
LMOD2	Leiomodin 2	1.199	**1.626** *	−1.126
CA3	Carbonic anhydrase III	1.252	**1.592** *	−1.269
MTRNR2L3	MT‐RNR2‐like 3	1.373	**1.503**	−1.005
HBA1	Hemoglobin, alpha 1	1.028	2.222	**2.135**
HBA2	Hemoglobin, alpha 2	−1.058	2.228	**1.715**
Genes down‐regulated relative to PRE				
DCN	Decorin	1.886	1.548	−**2.439** *
CLIC1	Chloride intracellular channel 1	1.218	1.180	−**2.089** *
S100A11	S100 calcium‐binding protein A11	1.013	1.275	−**2.037** *
RAMP2	Receptor (G protein‐coupled) activity‐modifying protein	1.126	1.305	−**1.926** *
S100A10	S100 calcium‐binding protein A10	1.089	1.035	−**1.918** *
TIMP3	TIMP metallopeptidase inhibitor 3	1.350	1.454	−**1.845** *
C1S	Complement component 1, s subcomponent	1.857	1.533	−**1.839** *
B2M	Beta‐2 microglobulin	1.494	1.161	−**1.790** *
MTIF3	Mitochondrial translation initiation factor 3	1.188	1.002	−**1.744** *
SPARCL1	SPARC‐like 1	1.616	1.247	−**1.722** *
TUBA1A	Tubulin alpha 1a	1.337	1.032	−**1.703** *
VIM	Vimentin	1.120	1.201	−**1.652** *
WDR5B	WD repeat domain 5B	1.546	1.198	−**1.643** *
PPIA	Peptidylprolyl isomerase A	1.262	1.071	−**1.629** *
EIF4E2	Eukaryotic translation initiation factor 4E family member 2	1.318	1.325	−**1.612** *
VEGFA	Vascular endothelial growth factor A	2.113	1.432	−**1.603** *
PSENEN	Presenilin enhancer gamma secretase subunit	1.408	1.198	−**1.581** *
TOMM6	Translocase of outer mitochondrial membrane 6 homolog (yeast)	1.202	1.206	−**1.539** *
ARL6IP1	ADP ribosylation factor like GTPase 6 interacting protein 1	1.769	1.651	−**1.528** *
OXSM	3‐oxacyl‐ACP synthase, mitochondrial	1.673	1.231	−**1.517** *
RPL17	Ribosomal protein L17	1.446	1.111	−**1.516** *

Values presented are mean fold‐change from pretreatment (PRE). LP‐PEPC, low‐pressure peristaltic pulse external pneumatic compression; MP‐PEPC, moderate pressure PEPC; *P <* *0.01 for within‐group Student's paired *t*‐test (bold added for emphasis); gene filtering and statistical analysis methods for gene expression changes are detailed in the Methods section of the manuscript.

Functional analyses revealed two statistically significant biological processes affected in the LP‐PEPC group at the POST1 time point, “cell morphology” (molecule involved: ARRDC2) and “cellular movement” (molecule involved: CA3). In the MP‐PEPC group at the POST1 time point, the top five biological processes significantly affected included “cellular movement” (molecules involved: DCN, S100A10, S100A11, SPARCL1, TUBA1A, PPIA TIMP3, VEGFA, and VIM), “cell‐to‐cell signaling and interaction” (molecules involved: DCN, PPIA, RAPM2, S100A10, TIMP3, VEGFA, and VIM), “cellular assembly and organization” (molecules involved: B2M, OXSM, PPIA, RAMP2, S100A10, SPARCL1, TIMP3, VEGFA, and VIM), “cellular function and maintenance” (molecules involved: B2M, DCN, OXSM, PPIA, S100A10, TIMP3, VEGFA, and VIM,), and “molecular transport” (molecules involved: B2M, DCN, HBA1/HBA2, OXSM, PPIA, S100A10, TIMP3, VEGFA, and VIM).

**Figure 1:**
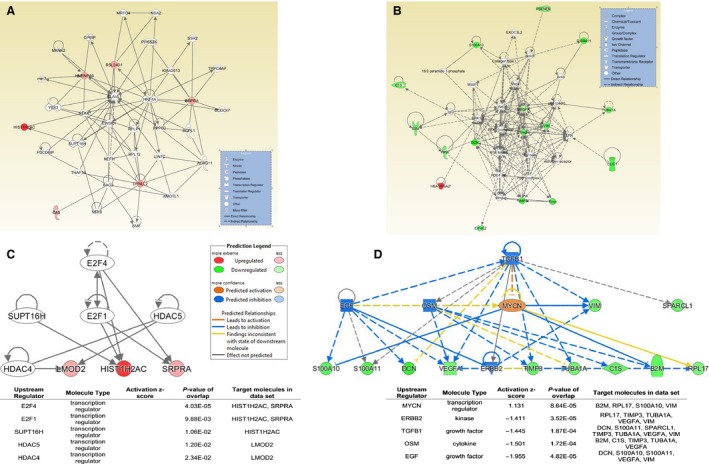
Ingenuity Pathway Analysis (IPA) using the genes significantly affected at the POST1 time point by low‐pressure peristaltic pulse external pneumatic compression (LP‐PEPC) and moderate pressure (MP‐PEPC). (A) Top gene network at POST1 for LP‐PEPC, (B) top gene network at POST1 for MP‐PEPC, (C) summary of upstream regulator analysis in IPA (upstream molecules that are causally connected to a subset of genes) at POST1 for LP‐PEPC, and (D) summary of upstream regulator analysis in IPA at POST1 for MP‐PEPC. For the upstream regulator analysis, the top five regulators are presented.

Figure 1A and B illustrate the top gene network generated by IPA based on the list of genes identified as significantly affected at POST1 in the LP‐PEPC and MP‐PEPC groups, respectively. For the LP‐PEPC group at the POST1 time point, the top gene network generated by IPA was “cancer, cellular movement, organismal injury and abnormalities” with a score of 15. For the MP‐PEPC group at the POST1 time point, the top gene network generated by IPA was “cancer, organismal injury and abnormalities, respiratory disease” with a score of 36.

Figure 1C and D show the results of Upstream Regulator Analysis in IPA for each of the top five regulators, along with overlapping *P*‐values and the number of downstream targets in the data set. Activation scores are given only for regulators that overlap with more than two genes in the data set.

A full list of significantly altered genes relative to PRE across all conditions at the POST2 time point is presented in Table 3. Only one gene was found to be significantly altered in the sham condition at the POST2 time point (up‐regulation of* LYPLA2*). There were 38 genes found to be significantly altered in the LP‐PEPC at POST2 with only one of those genes being down‐regulated (*TIMM10*). There were no significantly affected genes detected in the MP‐PEPC group at POST2.

**Table 3:** Relative changes in gene expression from PRE following seven consecutive days of sham, low pressure (LP‐PEPC; 30–40 mmHg), and moderate pressure (MP‐PEPC; 70–80 mmHg) peristaltic pulse external pneumatic compression (POST2 time point)

Gene	Description	Fold‐change from PRE		
		Sham	LP‐PEPC	MP‐PEPC
Genes up‐regulated relative to PRE				
LYPLA2	Lysophospholipase II	**1.515** *	−1.082	1.344
MYH2	Myosin, heavy chain 2, skeletal muscle, adult	1.235	**3.249** *	−1.084
ARRDC2	Arrestin domain containing 2	1.207	**2.898** *	1.567
IGFBP5	Insulin‐like growth factor‐binding protein 5	1.045	**2.846** *	−1.347
SYNM	Synemin	−1.129	**2.395** *	−1.190
DYNLL2	Dynein, light chain, LC8‐type 2	−1.074	**2.390** *	−1.182
MYOM1	Myomesin 1	−1.098	**2.356** *	1.029
MYH13	Myosin, heavy chain 13, skeletal muscle	1.283	**2.350** *	−1.180
BCL6	B‐cell CLL/lymphoma 6	1.350	**2.268** *	−1.343
TRDN	Triadin	−1.239	**2.145** *	−1.062
HNRNPA2B1	Heterogeneous nuclear ribonucleoprotein A2/B1	1.009	**2.041** *	−1.199
HNRNPUL2	Heterogeneous nuclear ribonucleoprotein U‐like 2	−1.090	**2.011** *	−1.217
GGT7	gamma‐glutamyltransferase 7	1.313	**1.999** *	−1.379
MYOZ3	Myozenin 3	1.023	**1.953** *	−1.225
NICN1	Nicolin 1	−1.030	**1.899** *	−1.210
MTIF2	Mitochondrial translation initiation factor 2	−1.175	**1.893** *	−1.028
CALCOCO1	Calcium binding and coiled‐coil domain 1	1.103	**1.854** *	−1.240
CSTF2T	Cleavage stimulation factor, 3′ pre‐RNA subunit 2, tau variant	1.014	**1.844** *	−1.350
C15orf52	Chromosome 15 open reading frame	1.193	**1.780** *	−1.289
MAFB	v‐maf avian musculoaponeurotic fibrosarcoma oncogene homolog B	1.325	**1.765** *	1.174
SRRM2	Serine/arginine repetitive matrix 2	1.138	**1.764** *	−1.253
ETFDH	Electron transfer flavoprotein dehydrogenase	−1.104	**1.762** *	−1.138
MAP4	Microtubule‐associated protein 4	−1.049	**1.760** *	−1.222
RFX5	Regulatory factor X5	1.215	**1.740** *	−1.044
PGK1	Phosphoglycerate kinase	1.117	**1.714** *	−1.134
GOT2	Glutamic‐oxaloacetic transaminase 2	−1.026	**1.686** *	1.037
C10orf71	Chromosome 10 open reading frame 71	1.095	**1.669** *	−1.107
SVIL	Supervillin	−1.154	**1.651** *	−1.083
PTCD3	Pentatricopeptide repeat domain 3	−1.090	**1.634** *	−1.014
SUOX	Sulfite oxidase	1.305	**1.633** *	−1.007
NFIX	Nuclear factor I/X (CCAAT‐binding transcription factor)	1.097	**1.625** *	−1.117
FAM53C	Family with sequence similarity 53 member C	1.072	**1.597** *	−1.186
MFN2	Mitofusin 2	1.007	**1.594** *	−1.037
SDHC	Succinate dehydrogenase complex subunit C	−1.042	**1.589** *	−1.165
HIST2H2BE	Histone cluster 2, H2be	−1.019	**1.557** *	−1.027
VEGFA	Vascular endothelial growth factor A	1.082	**1.508** *	−1.299
CS	Citrate synthase	−1.122	**1.505** *	−1.100
CAT	Catalase	−1.082	**1.500** *	−1.257
Genes down‐regulated relative to PRE				
TIMM10	Translocase of inner mitochondrial membrane 10 homolog (yeast)	1.434	−**1.513**	1.027

Values presented are mean fold‐change from pretreatment (PRE). LP‐PEPC, low‐pressure peristaltic pulse external pneumatic compression; MP‐PEPC, moderate pressure PEPC; *P <* *0.01 for within‐group Student's paired *t*‐test (bold added for emphasis); gene filtering and statistical analysis methods for gene expression changes are detailed in the Methods section of the manuscript.

In the LP‐PEPC group at the POST2 time point, the top five biological processes revealed by functional analysis to be significantly affected included “cellular development” (molecules involved: BCL6, CAT, HNRNPA2B1, IGFBP5, MAFB, MFN2, NFIX, PIP4K2B, and VEGFA), “cell growth and proliferation” (molecules involved: BCL6, CAT, HNRNPA2B1, IGFBP5, MAFB, MFN2, NFIX, PIP4K2B, and VEGFA), “free radical scavenging” (molecules involved: CAT. MFN2, SDHC, VEGFA), “molecular transport” (molecules involved: CAT, MFN2, SDHC, and VEGFA), and “cell death and survival” (molecules involved: BCL6, CAT, CS, HNRNPUL2, IGFBP5, MFN2, NFIX, SDHC, SVIL, SYNM, and VEGFA). Given the lack of significantly affected genes at the POST2 time point in the MP‐PEPC group, no significantly altered biological processes were identified.

**Figure 2:**
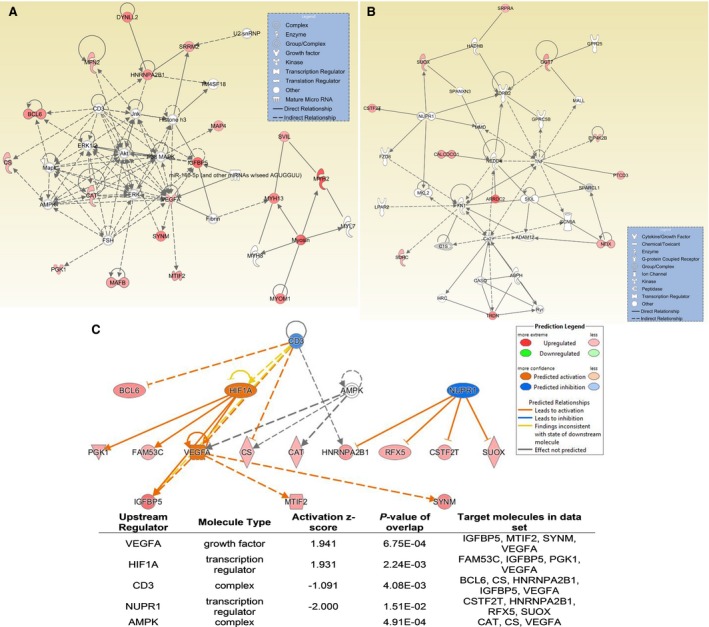
Ingenuity Pathway Analysis (IPA) using the genes significantly affected at the POST2 time point by low‐pressure peristaltic pulse external pneumatic compression (LP‐PEPC). (A) Top gene network at POST2 for LP‐PEPC, (B) second highest scored gene network at POST2 for LP‐PEPC, and (C) summary of upstream regulator analysis in IPA (upstream molecules that are causally connected to a subset of genes) at POST2 for LP‐PEPC. For the upstream regulator analysis, the top five regulators are presented.

Figure 2A and B illustrate the top two gene networks generated by IPA based on the list of genes identified as significantly affected at POST2 in the LP‐PEPC group. The top gene network generated by IPA was “cancer, hematological disease, immunological disease” with a score of 43 (Fig. 2A). The second highest scored gene network in the LP‐PEPC group at the POST2 time point was “cardiovascular disease, organismal injury and abnormalities, reproductive system disease” with a score of 23 (Fig. 2B). For the MP‐PEPC group at the POST1 time point, the top gene network generated by IPA was “cancer, hematological diseases, immunological disease” with a score of 43. Given the lack of significantly affected genes at the POST2 time point in the MP‐PEPC group, no gene networks were identified.

Figure 2C shows the results of Upstream Regulator Analysis in IPA for each of the top five regulators, along with overlapping *P*‐values and the number of downstream targets in the data set for the LP‐PEPC group at the POST2 time point. Activation scores are given only for regulators that overlap with more than two genes in the data set.

Interestingly, no genes were found to be commonly altered by both conditions (i.e., LP‐and MP‐PEPC groups) at either the singular POST1 or POST2 time points. However, the genes *ARRDC2* and *DYNLL2* were both found to be significantly up‐regulated in the LP‐PEPC group at the POST1 and POST2 time points. In addition, *VEGFA* gene expression was found to be significantly down‐regulated in the MP‐PEPC group at POST1, but significantly up‐regulated in the LP‐PEPC group at POST2.

### Protein expression with LP‐PEPC and MP‐PEPC

**Figure 3:**
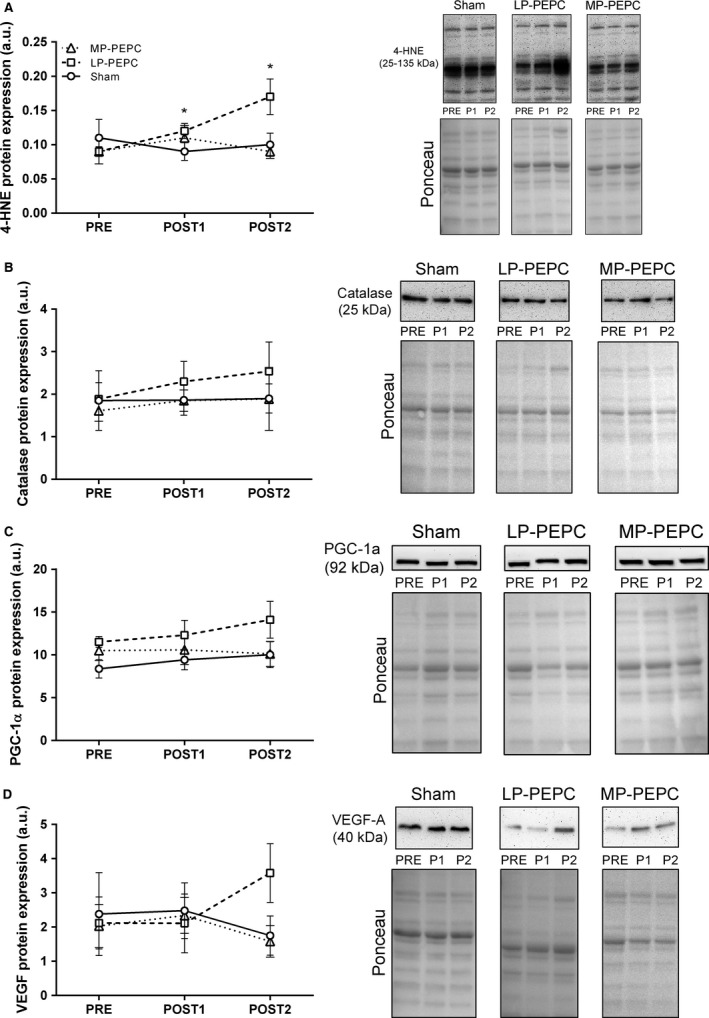
Effects of acute (POST1) and subchronic (POST2) peristaltic pulse external pneumatic compression (PEPC) and sham on selected skeletal muscle protein expression relative to pretreatment (PRE). (A) 4‐hydroxynonenal (4HNE), (B) catalase, (C) proliferator‐activated receptor gamma coactivator‐1 alpha (PGC‐1*α*), and (D) vascular endothelial growth factor A (VEGF‐A). A representative western blot image of all protein levels and respective Ponceau images are presented immediately to the right of each graph. LP, low pressure (30–40 mmHg); MP, moderate pressure (70–80 mmHg); P1, POST1; P2, POST2. All data are expressed as fold‐change from PRE levels (mean ± SEM, *n *= 5–6 subjects per target). Significance from between time points comparisons using Student's *t*‐tests are indicated within each panel. *significantly different from PRE in LP‐PEPC group.

Select protein expression was assessed in skeletal muscle biopsies respective to observed changes in gene expression. For p‐AMPK, no significant main effect of time nor group × time interaction was observed (*P *>* *0.05). Expression of p‐AMPK was 0.26 ± 0.12, 0.27 ± 0.07, and 0.24 ± 0.09 in the sham group, 0.35 ± 0.05, 0.31 ± 0.5, and 0.43 ± 0.08 in the LP‐PEPC group, and 0.31 ± 0.06, 0.28 ± 0.9, and 0.33 ± 0.07 in the MP‐PEPC group, at the PRE, POST1, and POST2 time points, respectively. No significant main effect of time was observed for 4HNE, CAT, PGC‐1*α*, or VEGF (Fig. 3). However, a time × group interaction was observed for 4HNE (*P *=* *0.014) and VEGF (*P *=* *0.035) protein expression (Fig. 1A and D). For 4HNE, expression was significantly greater at the POST1 and POST2 time points relative to PRE in the LP‐PEPC group (*P *=* *0.009, ES = 1.668 and *P *=* *0.020, ES = 1.258 for POST1 and POST2, respectively; Fig. 3A). In the MP‐PEPC group, change from PRE in 4HNE expression did not reach statistical significance at the POST1 (*P *=* *0.215, ES = 0.657) or POST2 (*P *=* *0.803, ES = 0.119) time points. For VEGF, expression was higher at the POST2 time point relative to PRE in the LP‐PEPC group, but did not reach statistical significance (*P *=* *0.045, ES = 1.282).

### Localization of the response

**Figure 4:**
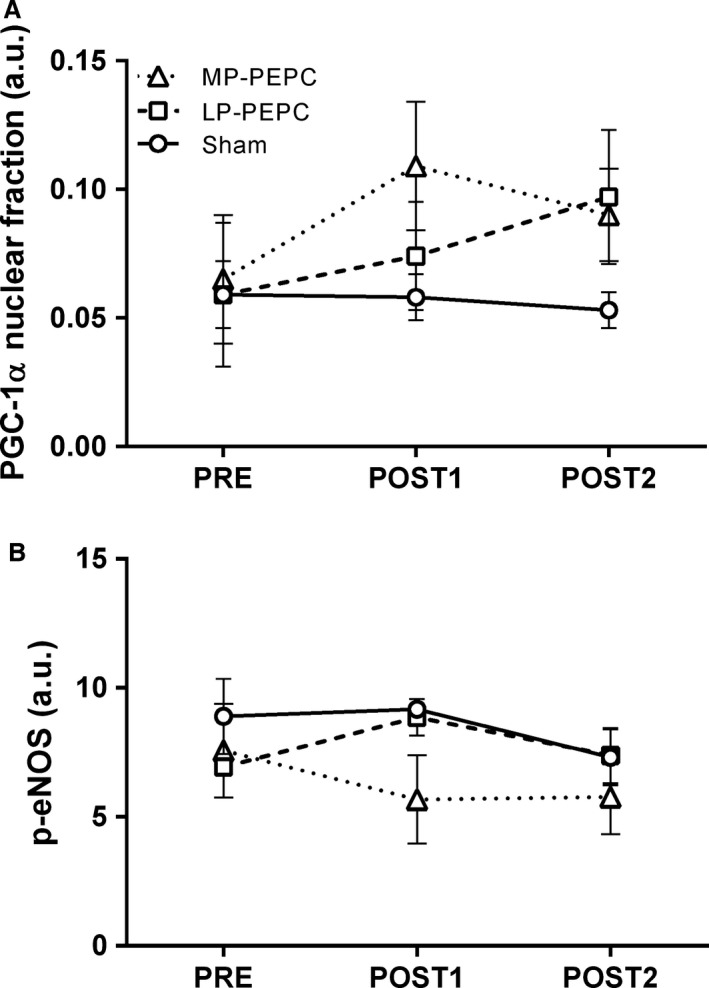
Effects of acute (POST1) and subchronic (POST2) peristaltic pulse external pneumatic compression (PEPC) and sham on (A) nuclear fraction of PGC‐1*α* and (B) phosphorylated eNOS (p‐ENOS) expression relative to pretreatment (PRE). Representative photomicrographs are presented in Figures 3 and 4. LP, low pressure (30–40 mmHg); MP, moderate pressure (70–80 mmHg); P1, POST1; P2, POST2. All data are expressed as fold‐change from PRE levels (mean ± SEM, *n *= 5–6 subjects per target).

**Figure 5:**
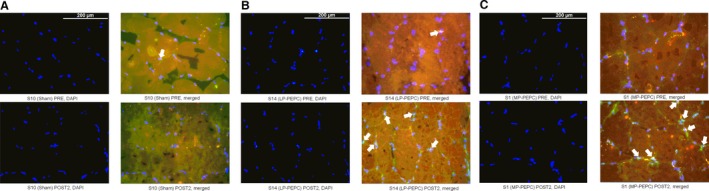
Representative 40x objective cross‐sectional images of nuclear fraction of PGC‐1*α* in *vastus lateralis* skeletal muscle biopsies pretreatment (PRE) and 24 h following seven consecutive days of treatment (POST2) with sham, low‐pressure peristaltic pulse external pneumatic compression (LP‐PEPC), and moderate pressure PEPC (MP‐PEPC). Slides were stained with antibodies against PGC‐1*α* and MAB and were counterstained with DAPI. Arrows indicate examples of identified PGC‐1*α* localized to the nucleus within the photomicrograph at the PRE and POST2 time points for (A) sham, (B) LP‐PEPC, and (C) MP‐PEPC.

**Figure 6:**
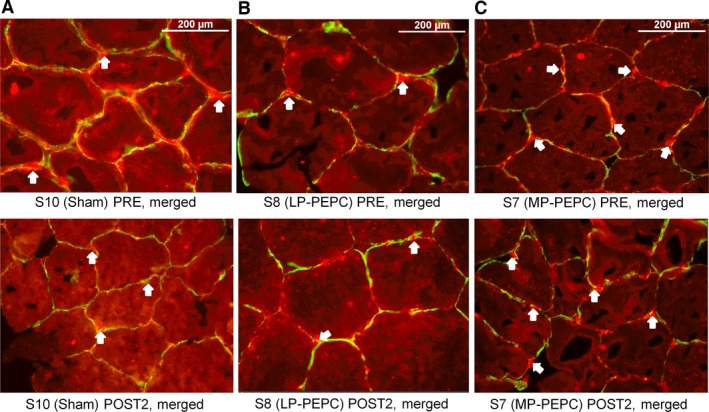
Representative 40x objective cross‐sectional images of phosphorylated eNOS in *vastus lateralis* skeletal muscle biopsies pretreatment (PRE) and 24 h following seven consecutive days of treatment (POST2) with sham, low‐pressure peristaltic pulse external pneumatic compression (LP‐PEPC), and moderate pressure PEPC (MP‐PEPC). Slides were stained with antibodies against phospho‐eNOS (SER1177) and dystrophin. Arrows indicate examples of identified phospho‐eNOS within the photomicrograph at the PRE and POST2 time points for (A) sham, (B) LP‐PEPC, and (C) MP‐PEPC.

In an effort to better assess the localization of the cellular response to PEPC, PGC‐1*α* localization within the nucleus and phosphorylation of capillary eNOS was assessed via immunohistochemistry. For nuclear fraction of PGC‐1*α*, there was no significant main effect of time (*P *=* *0.143) or time × group interaction (*P *=* *0.245) (Figs. 4A and 5). However, in the MP‐PEPC group, large effect sizes were observed relative to the change from PRE at the POST1 (ES = 0.995) and POST2 (ES = 0.910) time points. Similarly, no significant main effect of time (*P *=* *0.504) or time × group interaction (*P *=* *0.623) was observed for capillary phospho‐eNOS expression, although a large effect size (ES = 2.84) was observed in the LP‐PEPC group relative to PRE at the POST1 time point (Figs. 4B and 6).

### Citrate synthase activity

Citrate synthase activity did not vary between treatment groups at study entry (0.20 ± 0.04 vs. 0.17 ± 0.02 vs. 0.21 ± 0.06 mmol/L/min/mg for sham, LP‐PEPC, and MP‐PEPC, respectively). Moreover, no significant main effects of time, condition, or an interaction were found (*P *>* *0.05).

## Discussion

The primary findings of this study are that (1) independent of target inflation pressure, PEPC markedly affected *vastus lateralis* skeletal muscle biopsy gene expression 1 h following a single treatment and (2) there is significant heterogeneity in the gene expression response secondary to both time (i.e., timing posttreatment and/or acute vs. subchronic treatments) and target inflation pressures.

Of the 2566 genes meeting initial filtering criteria, only three (<1%) were found to change significantly within the sham group suggesting that our filtering and analysis methods were stringent enough to limit spurious findings and provide a relatively effective sham comparison group. Interestingly, *vastus lateralis* biopsies obtained 1 h following a single 1‐h PEPC application was shown to only up‐regulate gene expression in the LP‐PEPC group, but mostly down‐regulate gene expression in the MP‐PEPC group. Exceptions in the MP‐PEPC group included the up‐regulation of *HBA1* and *HBA2* (hemoglobin subunit alpha 1 and 2, respectively). The observed increase in mRNA levels of these two alpha‐globin genes could suggest a consistently higher proportion of erythrocytes in our skeletal muscle biopsy samples compared to PRE mediated by MP‐PEPC given that this mRNA is highly expressed in human red blood cells (Kabanova et al. 2009). Interestingly, oxidative stress has also been implicated in up‐regulation of hemoglobin in nonerythrocytes (Liu et al. 2011). Previously, we have observed modest increases in gene expression (e.g., *SOD2* mRNA), protein concentration (e.g., SOD2 protein), and metabolite concentrations (e.g., nitrate and nitrite) associated with redox signaling in *vastus lateralis* biopsy samples 1 and 4 h following application of a single PEPC treatment with only slightly lower target inflation pressures than those utilized for the MP‐PEPC group herein (60–70 mmHg) (Kephart et al. 2015). However, in this study, we did not observe a statistically significant change in markers of oxidized protein (i.e., 4HNE) or alteration in redox balance‐associated genes in the MP‐PEPC group (Fig. 3A).

*AARDC2* was among the up‐regulated genes in the LP‐PEPC group at the POST1 time point and was notable as it was also up‐regulated at the POST2 time point in this group. ARRDC2 is part of the *α*‐arrestin family of proteins involved with maintenance of optimal quantity and function of cell surface proteins, such as G protein‐coupled receptors (GPCR) and Notch receptors via kinase signaling and/or lysosomal activity according to cellular demands/stress (Shea et al. 2012). Notably, both GPCR and Notch are mechanosensitive (Morrow et al. 2005; Storch et al. 2012; Gordon et al. 2015) and it is not unreasonable to assume that mechanical stress is realized during PEPC treatment. Thus, *ARRDC2* up‐regulation may be in response to mechanical stress‐mediated alterations in GPCR activity and/or in acting to promote Notch activation via receptor proteolysis. Indeed, AARDC2 has been shown to interact, nonexclusively, with HECT ubiquitin ligases (i.e., WWP1, Nedd4), endosomal sorting complexes required for transport (ESCRT) components (i.e., ALIX) and ubiquitin (Rauch and Martin‐Serrano 2011). However, it is interesting that no significant alteration of *ARRDC2* was found in the MP‐PEPC group suggesting that the magnitude of mechanical stress and/or extracellular pressure is an important determinant of the response. As an alternative to mechano‐stress, ARRDC2 has been shown to be up‐regulated with corticosterone, though the effects of PEPC on circulating glucocorticoids are unknown (Guarnieri et al. 2012).

Similar to *AARDC2*,* DYNLL2* was found to be up‐regulated at both the POST1 and POST2 time points in the LP‐PEPC group. DYNLL2 is a component of the motor protein complex that has been linked to synaptic transmission reorganization in nerve cells and alteration/maintenance of spatial distribution of cytoskeletal structures (Suyama et al. 2016). However, at present, there is a paucity of literature examining the role of DYNLL2 in human skeletal muscle tissue, though it is plausible that up‐regulation of this gene is related to maintenance of cellular homeostasis and neural synapses following mechanical disruption/stress of the nerve and/or muscle cells.

With regard to the transcriptome‐wide responses to PEPC, it was remarkable to observe that at both the POST1 and POST2 time points, nearly all significantly altered genes in the LP‐PEPC group (98%) were up‐regulated, while nearly all of the significantly altered genes in the MP‐PEPC group (91%) were down‐regulated. Moreover, the strong majority (96%) of the significantly affected genes were isolated to a specific condition (LP‐ vs. MP‐PEPC) and time point (i.e., POST1 vs. POST2) further illustrating the marked heterogeneity of the responses observed herein. While the absolute difference in PEPC inflation pressures is modest (40 mmHg), the role of pressure in the observed gene expression response is intriguing. Evidence from blood flow restriction training studies in subjects of similar age and body size (i.e., BMI) would suggest that complete arterial blood flow occlusion is unlikely to occur with maximal pressures of 70–80 mmHg (Laurentino et al. 2012). However, wider cuffs achieve occlusion at lower pressures (Scott et al. 2015) and with PEPC, generally more than one segment of the “leg sleeve” is inflated at time creating a compressed area much greater than that traditionally applied with inflatable cuffs in blood flow restriction training. To examine the role of target inflation pressures, we conducted a follow‐up analysis evaluating femoral artery blood flow for an entire compression cycle (i.e., zones 1–5 and deflation periods) compared to rest (Fig. S1). In this follow‐up study, we found that the mean femoral artery blood flow area under the curve was significantly decreased in the MP‐PEPC condition for the period comprising compression and remained significantly decreased when considering (i.e., including in AUC analysis) the deflation period between cycles. Mean femoral artery blood flow was also depressed in the LP‐PEPC condition, though it did not reach statistical significance (*P *>* *0.200). Though this was only a small follow‐up study, at a minimum, it is reasonable to conclude that, on average, there is at least partial occlusion and/or increased resistance to arterial inflow, but to a greater degree in the MP‐PEPC group. With respect to venous outflow, the MP‐PEPC group is certain to realize venous outflow occlusion when devoid of hypertension, whereas the LP‐PEPC group may or may not have total occlusion of venous outflow (Groothuis et al. 1985). Again, given the “cuff” sizes utilized with PEPC, as well as the peristaltic nature of the compressive stimulus, it is more likely than not that there is occlusion of venous outflow even in the LP‐PEPC group. Therefore, arterial inflow during PEPC treatment may be one explanation for observed heterogeneity in the response to PEPC.

Between‐subject differences in arterial blood pressure and limb circumference(s) may also have a profound impact on the response to PEPC. Indeed, if arterial inflow disruption during PEPC contributes to the cellular response observed, then an individual's resting arterial blood pressure should be considered. Loenneke et al. have previously demonstrated that peripheral systolic blood pressure is a significant predictor of the magnitude of arterial occlusion with a given occlusion cuff pressure (Loenneke et al. 2015). In addition, this group also found that limb circumference was a significant, and even stronger, predictor of the degree of arterial occlusion (Loenneke et al. 2015). Thus, limb circumference, in addition to blood pressure, may also have a significant effect on the degree of arterial occlusion realized with standardized PEPC target inflation pressures. Moreover, it is reasonable to assume similar variability in the alteration of intramuscular pressures and/or mechanical stress mediated by set PEPC target inflation pressures. In hindsight, participant blood pressure and limb circumference characteristics may have provided further clarity in the PEPC‐mediated changes in skeletal muscle tissue. Unfortunately, we were unable to obtain a suitable portion of these characteristics for various reasons (e.g., loss of contact with subject, relocation, etc.). Regardless, we have previously seen PEPC‐mediated up‐regulation of skeletal muscle genes with nearly the same pressure (i.e., 60–70 mmHg) as that utilized in our MP‐PEPC group (i.e., 70–80 mmHg) and others have reported up‐regulation with similar interventions (i.e., IPC) with pressure magnitudes greater than those used herein (Sheldon et al. 2012; Kephart et al. 2015). Thus, our findings were surprising and warrant further investigation with (1) a wider range of target inflation pressures and (2) a particular emphasis on standardizing “realized” intramuscular and/or arterial occlusion pressure while accounting for individuals’ characteristics (e.g., blood pressure, limb circumference).

*VEGFA* gene expression, well known to be up‐regulated in response to hypoxia (or low oxygen tension) (Ferrara and Davis‐Smyth 1997), was found to be up‐regulated at the POST2 time point following subchronic PEPC treatment in the LP‐PEPC group, but down‐regulated acutely at the POST1 time point in the MP‐PEPC group. Moreover, VEGF protein tended to be increased at POST2 in the LP‐PEPC group, but not in the MP‐PEPC group (Fig. 3D). This paradoxical between‐group finding was surprising given the alterations in local blood flow and shear stress with the respective PEPC compressive pressures. However, *VEGFA* has also been shown to be up‐regulated in response to cytokines, redox stress, hypoxia, and/or cell differentiation/transformation (Ferrara and Davis‐Smyth 1997). At present, the acute and subchronic effects of PEPC on cytokines and cell differentiation have not been characterized, though we have previously demonstrated a significant decrease in *HIF*‐*1α* mRNA 4 h following an acute bout of PEPC with target inflation pressures of 60–70 mmHg (Kephart et al. 2015). Moreover, in this study, IPA analysis suggests significant upstream up‐regulation of HIF‐1*α* and VEGFA evidenced by the respective alterations in the expression of *FAM53C*,* IGFBP5*,* MTIF2*,* PGK1*, and *SYNM* genes at the POST2 time point in the LP‐PEPC group. Thus, the results herein suggest that, specific to LP‐PEPC group, there may have been a greater stimulus for the vasculature and/or *VEGF* mRNA expression and translation. Indeed, we did observe a very large effect size (ES = 2.84) relative to phosphorylation state of capillary eNOS acutely in the LP‐PEPC group, but not in the MP‐PEPC group (Fig. 4B).

Interestingly, despite primarily down‐regulation of gene expression at POST1 and limited alteration of gene expression at POST2 in the MP‐PEPC group, large effect sizes (ES > 0.9) were observed for fraction of PGC‐1*α* localized to the nucleus. This particular measure was of interest to our group given that we have previously noted up‐regulation of *PGC*‐*1α* mRNA with an acute bout of PEPC and because PGC‐1*α* translocation to the nucleus may suggest that the skeletal muscle fiber itself (as opposed to nerves, vessels, etc. contained within biopsy sample) is indeed responsive to the PEPC‐mediated “stress”. Although we did not observe a statistically significant group × time interaction relative to nuclear fraction of PGC‐1*α*, the statistical power for western blot and immunohistochemistry investigations was quite low. The primary aim of this study was to characterize transcriptome‐wide changes in gene expression with PEPC and the number of subjects included was low (6/group) to prevent overpowering the study given the propensity to do so with RNA sequencing investigations (Fang and Cui 2011). Notably, though not reaching the threshold for a “large”, the observed effect size for PGC‐1*α* localization to the nucleus in the LP‐PEPC group was 0.232 and 0.650 for the POST1 and POST2 time points, respectively. Moreover, pooling all PEPC subjects results in effect sizes of 0.844 and 1.08 at the POST1 and POST2 time points, respectively, suggesting that PEPC, at these pressures in general, has a significant effect on PGC‐1*α* localization to the nucleus that may be more pronounced with higher target inflation pressures. In sum, the large effect sizes relative to nuclear fraction of PGC‐1*α* observed at POST1 and POST2 exclusively in the MP‐PEPC group combined with the large effect size relative to phosphorylated capillary eNOS at POST1 in the LP‐PEPC group suggests that higher pressure PEPC may have a greater effect on the muscle fiber itself, whereas lower pressure PEPC may more selectively target the vasculature.

Pertinent to this study, it is imperative to consider the timing of muscle biopsy sampling. The POST1 time point represented biopsy that occurred 1 h following a single bout of PEPC, whereas the POST2 time point represented biopsy that occurred 24 h following the seventh consecutive day of PEPC treatment. Our rationale for the timing was that that 1‐h post‐PEPC demonstrated an acute response to treatment at a time point that had previously demonstrated greater gene expression changes than 4 h post‐PEPC (Kephart et al. 2015). Moreover, we chose to perform the POST2 biopsies 24 h following the last treatment in an effort to capture the subchronic effects on gene expression devoid of acute gene expression changes relative to that last treatment session. Also, worthy of consideration is the fact that three skeletal muscle biopsies were taken from slightly different regions of the *vastus lateralis*. To this end, the methods employed were designed to limit inflammatory influence at the site of harvest and was standardized relative to distance from prior biopsies for all participants. Moreover, citrate synthase activity in the biopsy samples did not vary between time points suggesting that fiber‐type composition was relatively stable. Regardless, regional differences in biopsy composition cannot be excluded (Brutsaert et al. 2002).

Despite the aforementioned limitations, this is the first study to use a transcriptome‐wide approach in order to interrogate molecular alterations that occur in skeletal muscle in response to both on week of PEPC in humans. Given that we found significant heterogeneity in the gene response to PEPC with time (acute vs. subchronic) and with different target inflation pressures (LP‐ vs. MP‐PEPC), these data warrant further research with regard to how PEPC affects functional outcomes particularly in targeted clinical and/or athletic populations.

## Conflicts of Interest

NormaTec (Newton Center, MA) provided partial financial support (50%) of this project through a contract awarded to JSM. However, the authors have no conflicts of interest to disclose.

## Supporting information




**Figure S**1**.** Femoral artery blood flow during a complete cycle of PEPC at low (LP) and moderate (MP) target inflation pressures.
