# Supplementary material for: Impact of external pneumatic compression target inflation pressure on transcriptome‐wide RNA expression in skeletal muscle
Source: Physiol Rep. 2016 Nov 24;4(22):e13029. doi: 10.14814/phy2.13029 (PMC5357997; doi:10.14814/phy2.13029)
Supplement: Supplementary file 2 [file PHY2-4-13029-s002.docx]

Figure S1. Femoral artery blood flow during a complete cycle of PEPC at low (LP) and moderate (MP) target inflation pressures. Six (n=6) participants were recruited for participation in this crossover design follow-up analysis. Using high resolution ultrasound (Logiq S7 R2 Expert; General Electric, Fairfield, CT USA) with a 3 to 12 MHz multi-frequency linear phase array transducer the left common femoral artery was imaged longitudinally 2-3 cm proximal to the bifurcation. Simultaneous measurement of femoral artery diameter and blood velocity was performed using duplex mode imaging (B-mode and Doppler) and video was captured through a digital interface at 30 frames/s with real time analysis (FMD Studio, Pisa, Italy). Resting measurements were obtained for 30 seconds, imaging was paused and resumed with the start of peristaltic compression in zone 1. Thereafter, continuous measurement of diameter and blood velocity continued through each 30-seconds of compression of zones 2-5 and for the 30 seconds of complete deflation. Vessel diameters were determined frame-by-frame via automatic edge detection software (FMD Studio, Pisa, Italy) measuring the distance between the near and far wall of the intima. Blood velocity was determined via selection of a region of interest around the Doppler waveform and a trace of the velocity-time integral was used to calculate mean velocity for each cardiac cycle. Antegrade (panel A), retrograde (panel B), and mean (panel C) femoral artery blood flow was calculated from continuous diameter and blood velocity measurements during ultrasonography as [Π * (diameter/2)^2^ * time average mean velocity * 60]. Finally, mean femoral artery blood flow area under the curve was determined for the compression periods (only zones 1-5) as well as compression + deflation periods (zones 1-5 + deflation time) using the sum of trapezoids method after resting blood flow was subtracted (Panel D). For panels A-C, statistical analyses were performed using repeated measures ANOVA with post-hoc paired t-tests with Bonferroni correction for multiple comparisons (α=0.0083) employed when a significant condition*time interaction was observed. For panel D, one-sample t-tests with Bonferroni correction for multiple comparisons (α=0.0125) were used for statistical analysis. All data are presented as mean ± S.E.M. ϕ, significantly different from Rest in MP-PEPC group; †, significantly different between conditions at the same time point; *, significantly different from zero.
